# Multi-state modeling associates proliferation genes with early estrogen receptor-positive breast cancer recurrence and survival

**DOI:** 10.21203/rs.3.rs-10159953/v1

**Published:** 2026-06-30

**Authors:** Yongzhe Wang, Christine Quinones, Irene Kang, Hope Rugo, Ernest Martinez, Victoria Seewaldt, Joanne Mortimer, Aritro Nath, Veronica Jones

**Affiliations:** City of Hope National Medical Center; City of Hope National Medical Center; City of Hope National Medical Center; City of Hope National Medical Center; University of Riverside California; City of Hope National Medical Center; City of Hope National Medical Center; City of Hope National Medical Center; City of Hope National Medical Center

## Abstract

**Background:**

To identify baseline gene expression programs associated with recurrence timing in estrogen receptorpositive (ER+) breast cancer (BC) using a multi-state modeling framework.

**Methods:**

We analyzed high-risk ER + BC patients with surgical tumor specimens profiled using a custom NanoString panel (145 genes). Recurrence was classified as none, early (<5 years after surgery), or late (≥ 5 years). Differential gene expression was assessed using negative binomial regression. Transition-specific risks of recurrence and survival were estimated using semi-Markov multi-state Cox models, with sensitivity analyses conducted under alternative definitions of early and late recurrence.

**Results:**

Among 79 patients analyzed, 28% developed recurrence (14 early, 9 late), and all BC-specific deaths occurred following early recurrence. In transition-specific models, higher baseline expression of proliferation-related genes, including *UBE2C, EZH2, CCNB1, PTTG1*, and *MKI67*, was consistently associated with increased risk of early recurrence (hazard ratio [HR] range: 1.91–2.14; all p < 0.05), whereas *SNAI2* expression was protective (HR: 0.43; 95% CI: 0.18–0.99). In contrast, late recurrence was associated with signaling-related genes such as *IGF1R, LEF1*, and *WNT7B* (HR range: 1.52–3.40; all p < 0.05). Early recurrence-associated genes showed concordance across differential expression analyses and sensitivity analyses, supporting biological coherence.

**Conclusions:**

This study identifies a potential proliferative gene assay associated with early recurrence in ER + BC, while late recurrence appears linked to distinct signaling pathways. These findings prioritize candidate molecular biomarkers for future validation to improve risk stratification and surveillance.

## Background

Breast cancer (BC) remains the most frequently diagnosed malignancy and the second leading cause of cancer-related mortality in women in the United States (1). Estrogen receptor-positive breast cancer (ER + BC) represents approximately 70% of all breast cancer cases and, because of its high incidence, accounts for the majority of breast cancer-related deaths. The five-year survival rate for localized ER + BC remains high but declines sharply to 36.5% once the disease becomes metastatic (2). Although a subset of patients presents with de novo metastatic disease, the majority of metastatic progression arises from disease recurrence (3). Recurrence itself represents a major prognostic inflection point; compared with patients who remain disease-free, those who experience recurrence face a threefold higher risk of death, which rises to fivefold when recurrence occurs early (defined as relapse within five years of surgery) (4, 5). These differences highlight the profound molecular heterogeneity that characterizes ER + BC and its influence on clinical outcomes.

At the biological level, molecular heterogeneity in ER + BC was initially characterized by immunohistochemical profiling of estrogen receptor expression in tumor nuclei, leading to the delineation of intrinsic subtypes—luminal A, luminal B, HER2-enriched, and basal-like (6, 7). Numerous studies have demonstrated that luminal A tumors generally confer a favorable prognosis; luminal B tumors exhibit higher proliferation rates, increased endocrine therapy (ET) resistance, and worse outcomes; HER2-enriched tumors respond poorly to ET; and basal-like tumors behave more similarly to triple-negative breast cancer (6–10). Collectively, luminal lineages comprise approximately 60–70% of all breast cancer diagnoses (11–13) and display distinct genomic and epigenetic patterns compared with HER2-enriched and basal-like tumors (14, 15). Within the luminal spectrum, luminal A tumors typically show lower proliferative activity and later recurrence relative to luminal B (9). As ER + BC encompasses the majority of breast cancer cases and luminal B carries a greater recurrence risk than luminal A, these clinical differences are likely driven by underlying genomic and epigenomic variability.

Commercially available multigene assays, such as Oncotype DX, EndoPredict, PAM50/Prosigna, and Breast Cancer Index (BCI), have significantly advanced the prognostic evaluation of ER + BC by integrating gene expression profiles into composite recurrence scores (16–21). While these tools are clinically validated and widely adopted, their fixed proprietary algorithms aggregate gene signals into single risk indices, limiting interpretability at the individual gene level and obscuring the differential contribution of molecular processes to early (<5 years) versus late (≥ 5 years) recurrence. In this study, we analyzed a curated 128-gene panel comprising transcripts from these established assays and applied multi-state modeling to characterize how genes and biological pathways are associated with early and/or late recurrence, as well as post-recurrence survival. This gene-level analytic framework enhances the biological resolution of recurrence prediction, providing mechanistic insights that may inform ET selection and time-adaptive surveillance strategies. Ultimately, our study seeks to leverage a well-characterized ER + BC cohort with 10 years of longitudinal follow-up to bridge the gap between clinically validated prognostic assays and biologically interpretable recurrence mechanisms.

## Methods

### Study design and patient population

We identified ER+HER2– BC formalin-fixed paraffin-embedded specimens resected at a national cancer center (2012–2016) from surgical pathology archives to construct the retrospective cohort. Eligible female patients had provided informed consent (IRB 07047). Due to the retrospective nature of the data collection, attrition was not applicable and no randomization was performed. ER positivity was defined as ≥ 10% to exclude triple-negative-like tumors. Inclusion criteria required a primary tumor ≥ 2 cm or <2 cm with biopsy-confirmed nodal metastasis to select for high-risk disease, and ≥ 3 years of followup. Patients with metastatic disease at diagnosis were excluded. Recurrence was defined as locoregional or distant disease ≥ 3 months after definitive treatment, and recurrence status was not blinded to the researchers. A custom NanoString nCounter panel (145 genes: 128 endogenous, 17 housekeeping; NanoString Technologies, WA) was used to profile tumor samples collected at surgery and risk of recurrence scores were not generated (22).

### Statistical considerations

#### Descriptive analysis

BC recurrence was classified as no recurrence, early recurrence (<5 years), or late recurrence (≥ 5 years). Patient demographics were summarized by recurrence status, with group differences in categorical covariates evaluated using Fisher’s exact test. Differences in cause-specific cumulative incidence of death across recurrence groups were assessed using Gray’s test. An alluvial diagram was constructed to illustrate patient trajectories across recurrence and survival status. To explore patterns of molecular association, Pearson correlation heatmaps were generated for pairwise comparisons (no versus early recurrence, no versus late recurrence, and early versus late recurrence). For differential expression gene (DEG) analysis, volcano plots were used to visualize gene-level associations, and negative binomial regression was applied to estimate log2 fold changes in gene expression, adjusting for age at diagnosis, cancer stage, and tumor size.

### Semi-Markov multi-state analysis

For time-to-event analysis, we used a semi-Markov multi-state model to capture the natural history of recurrence and survival (23–25). In this framework, baseline transitions (from no recurrence to early recurrence, late recurrence, or death without recurrence) were modeled as functions of time since definitive surgery. Post-recurrence transitions (from early or late recurrence to BC-specific death or other-cause death) were modeled as functions of time since recurrence, reflecting the clinical relevance of sojourn time. By modeling recurrence as a sequence of clinically meaningful states rather than a single composite endpoint, we were able to isolate baseline molecular features specifically associated with progression into early/late recurrence. A state-transition diagram was generated to depict the model structure. Transition intensities were estimated using transition-specific (cause-specific) Cox proportional hazards models, with cluster-robust standard error and proportionality assessed by Schoenfeld residuals. For transitions from no recurrence to early or late recurrence, patients experiencing the alternative recurrence type were censored at the time of event to target cause-specific hazards, and models were adjusted for age, cancer stage, and tumor size. Continuous gene expressions were standardized, and hazard ratios were estimated per one standard deviation (SD) increase. To provide clinically interpretable absolute risk estimates under competing events, cumulative incidence functions were estimated using the Aalen-Johansen method for early recurrence, late recurrence, and pre-recurrence other-cause death, stratified by tertiles of gene expression.

Because some transitions involved sparse events (notably, other-cause death without recurrence and breast cancer death after early recurrence), we applied ridge-penalized Cox proportional hazards models to stabilize estimation (26–28). Ridge penalization shrinks regression coefficients toward zero without performing variable selection, thereby reducing variance while retaining all prespecified covariates in the model (27). Default penalty settings were used, and no data-driven tuning was performed. For transitions to other-cause death after early or late recurrence, only univariable Cox models for the gene of interest were fitted, as the number of observed events (n = 14 and n = 9, respectively) was insufficient to support multivariable adjustment even under penalization (29–31). To examine robustness to the classification of recurrence timing, sensitivity analyses were performed by redefining early versus late recurrence using 3-year and 7-year thresholds (32, 33).

### Pathway analysis

Beyond individual gene-level inference, pathway-level inference was investigated using both overrepresentation analysis (ORA) and gene set enrichment analysis (GSEA) for each transition setting (32–34). In ORA, hypergeometric tests were used to assess whether the subset of significant genes identified by transition-specific Cox models was over-represented in predefined functional categories, with p values adjusted using the Benjamini-Hochberg method. In GSEA, all genes were ranked by their association statistics, and enrichment scores were calculated using a weighted Kolmogorov-Smirnov–like statistic to evaluate whether pathway genes were disproportionately concentrated toward one end of the ranked list. All statistical tests were two-sided, with p <0.05 considered significant. Analyses were conducted in R version 4.1.1.

## Results

### Distribution of recurrence timing and deaths

**Figure 1:**
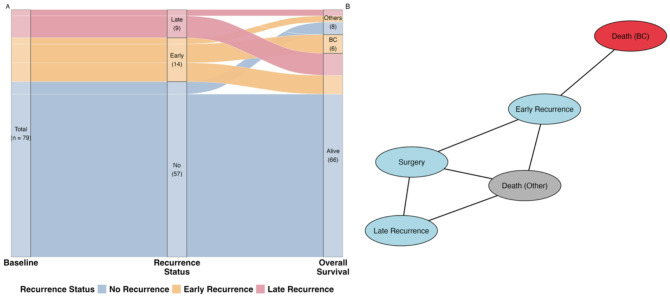
Patient trajectories and multi-state model structure. An alluvial diagram (A) shows trajectories of 79 patients from baseline through recurrence status (no recurrence, early recurrence within 5 years, and late recurrence ≥5 years after definitive surgery) to overall survival outcomes (alive, breast cancer death, other-cause death). A state-transition diagram (B) illustrates the semi-Markov modeling framework applied to evaluate recurrence and survival. BC, breast cancer.

The cohort included 79 ER + BC patients contributing 80 samples (one patient had bilateral tumors). Of these, 56 patients had no recurrence, 14 experienced recurrence (12 of 14 were metastatic recurrences) within 5 years after definitive surgery (early recurrence), and 9 experienced recurrence after 5 years (late recurrence), yielding an overall recurrence proportion of 28% (Table 1). Though all were ER+HER2- by immunohistochemical classification, by intrinsic subtype classification 49 patients were luminal A, 27 were luminal B, and 3 were HER2-enriched. 53% of patients were non-Hispanic White, and the majority (≥ 50%) were aged ≤ 65 years and had BMI ≤ 30 at diagnosis. Documented Oncotype DX recurrence score (RS) was available for 42 of 79 patients. Among patients with available scores, 21 were classified as low risk, 16 as intermediate risk, and 5 as high risk. Early-recurrence cases showed a numerically higher proportion of intermediate- or high-risk scores compared with patients without recurrence. All BC-specific deaths occurred among patients with early recurrence, representing a significant difference across recurrence groups (p <0.001). Deaths due to other causes included 4 patients without recurrence, 2 with early recurrence, and 2 with late recurrence (Fig. 1A). Using the 5-year cutoff for defining early versus late recurrence, no BC-specific deaths were observed after late recurrence with a mean follow-up of 8 years. Thus, patients without recurrence at baseline either subsequently developed recurrence (early or late) or died from other causes (Fig. 1B). No other significant group differences were observed.

### Baseline gene expression patterns by recurrence timing

Patterns of gene association revealed several clusters of highly correlated genes across the three pairwise comparisons (Figure S1). The most prominent was a tightly correlated module dominated by proliferation and cell-cycle genes (e.g., *MKI67*, *TOP2A*, *BRCA1*, *BIRC5*, *UBE2C*, *CCNB1*, *CDC20*, *MELK*, *EZH2*, *CENPF*, and *CDCA* family members), which was largely anti-correlated with a module comprising EMT-related and mesenchymal-state genes (e.g., *VIM*, *SNAI2*, *TWIST1*, *TWIST2*, *ZEB2*). Additional, smaller co-expressed clusters included basal/stem-associated cytokeratins (*KRT5*, *KRT14*, *KRT17*), immune-related genes (e.g., *CD8A*, *STAT1*, *CD27*), and growth-factor signaling components (e.g., *IGF1*, *EGFR*, *HGF*). Overall, the block structure of gene co-expression was largely preserved across the recurrence comparisons.

**Figure 2:**
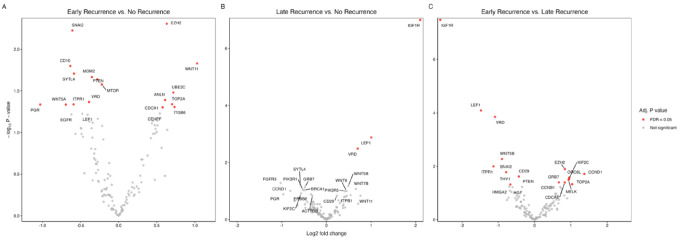
Volcano plots of differential gene expression analysis across recurrence types. Differentially expressed genes are shown for (A) early versus no recurrence, (B) late versus no recurrence, and (C) early versus late recurrence. The x-axis represents log2 fold change and the y-axis represents −log10 p-value. Abbreviations: Adj, adjusted; vs, versus.

In DEG analysis, early recurrence compared with no recurrence was associated with higher expression of *EZH2, WNT11, UBE2C, ANLN, TOP2A, CDCA1, RRM2, MELK, CENPF*, and *CCNB1* (p < 0.05, Fig. 2). Conversely, *SNAI2, SYTL4, PTEN, MTOR, VIM, ITPR1, CD10*, and *LEF1* were expressed at lower levels in early recurrence. When comparing early with late recurrence, higher expression of *IGF1R, LEF1, VRD*, and *WNT11* was associated with late recurrence, while *SYTL4* expression was consistently lower in recurrent cases regardless of recurrence type. Overall, early recurrence was characterized by relative enrichment of genes involved in cell-cycle regulation and proliferation (*EZH2, TOP2A, CCNB1, RRM2, MELK, CENPF*, and *CDCA* family members) and relative depletion of tumor suppressor and EMT/differentiation-related genes (*PTEN, LEF1, SNAI2, ITPR1*, and *VIM*). Effect-size patterns were directionally consistent across comparisons. Growth factor signaling genes (*IGF1R, HGF*) were expressed at lower levels in early compared with late recurrence, suggesting distinct baseline molecular profiles associated with recurrence timing.

### Transition-specific hazards: early and late recurrence

**Figure 3:**
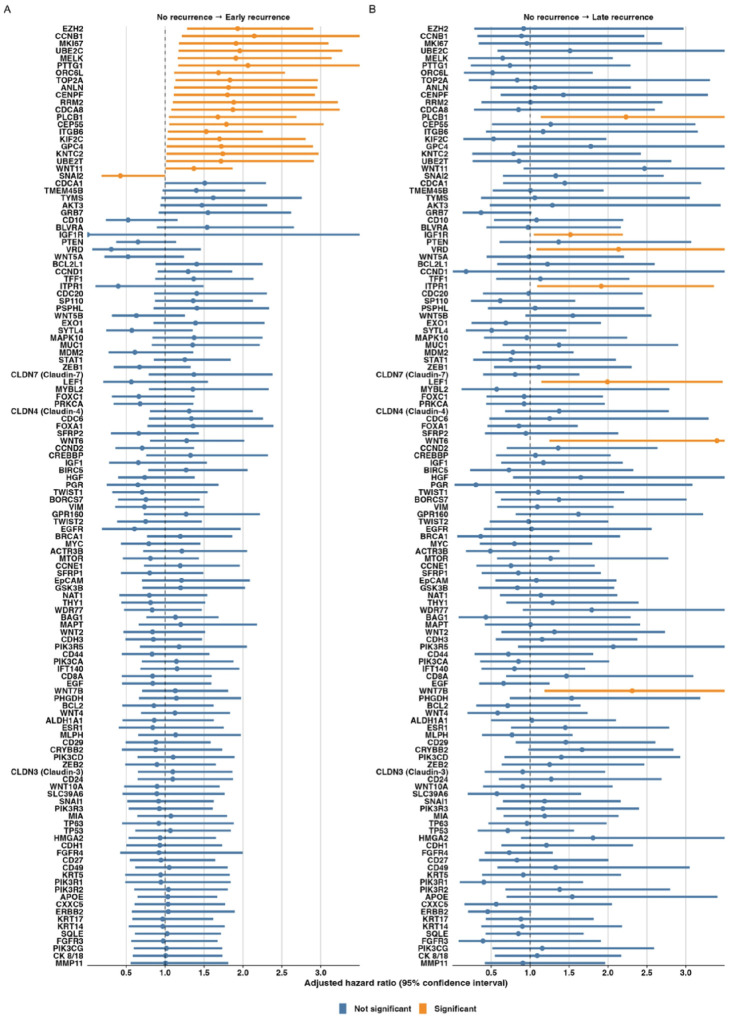
Forest plots of transition-specific Cox models for recurrence. Hazard ratios with 95% confidence intervals for 128 genes are shown for (A) the transition from no recurrence to early recurrence and (B) the transition from no recurrence to late recurrence.

To capture the temporal structure of recurrence and survival, transition-specific semi-Markov models were developed. In the transition from no recurrence to early recurrence, higher expression of 20 genes—including *EZH2, CCNB1, MKI67, UBE2C, MELK, PTTG1, ORC6L, TOP2A, ANLN, CENPF, RRM2, CDCA8, PLCB1, CEP55, ITGB6, KIF2C, GPC4, KNTC2, UBE2T*, and *WNT11*—was associated with increased risk of recurrence within 5 years after definitive surgery (HR range: 1.37–2.14, all p < 0.05, Fig. 3A). Among the top genes, a 1-SD increase in *CCNB1, PTTG1, UBE2C, EZH2*, and *MKI67* expression corresponded to 114% (HR: 2.14, 95%CI: 1.21–3.79), 105% (HR: 2.07, 95%CI: 1.17–3.64), 96% (HR: 1.96, 95%CI: 1.17–3.28), 93% (HR: 1.93, 95%CI: 1.28–2.91), and 91% (HR: 1.91, 95%CI: 1.18–3.10) higher risk of early recurrence, respectively (Table S1). In contrast, *SNAI2* expression was associated with lower risk of early recurrence (HR: 0.43, 95%CI: 0.18–0.99). Ten genes (*EZH2, CCNB1, UBE2C, MELK, TOP2A, ANLN, CENPF, RRM2, WNT11, SNAI2*) overlapped with those identified by DEG analysis comparing early versus no recurrence.

**Figure 4:**
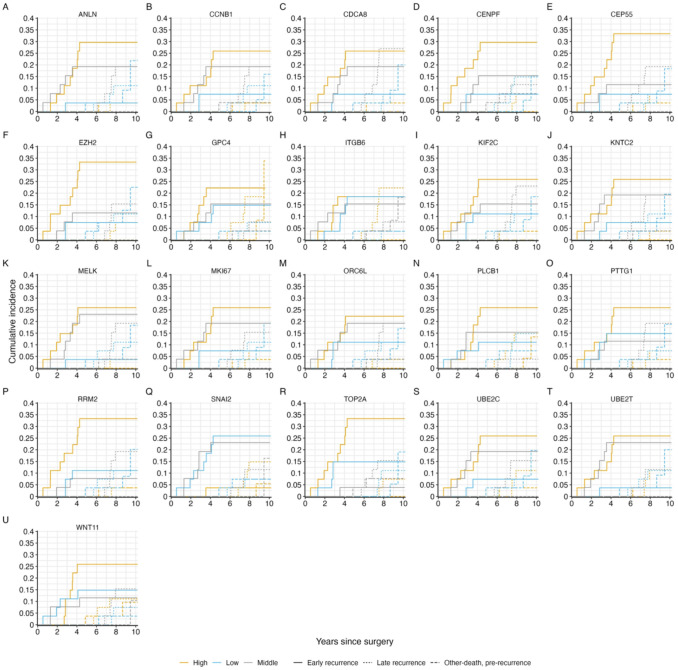
Cumulative incidence curves of recurrence and survival stratified by gene expression levels. Curves for early recurrence, late recurrence, and other-cause death without recurrence are shown by low (<33rd percentile), middle (33rd–66th percentile), and high (>66th percentile) expression levels. Twenty-one representative genes (A-U) significantly associated with early recurrence (*ANLN, CCNB1, CDCA8, CEP55, EZH2, GPC4, ITGB6, KIF2C, KNTC2, MELK, MKI67, ORC6L, PLCB1, PTTG1, RRM2, SNAI2, TOP2A, UBE2C, UBE2T,* and *WNT11*) are displayed from the full 128-gene panel.

In the transition from no recurrence to late recurrence, seven genes (*PLCB1, IGF1R, VRD, ITPR1, LEF1, WNT6*, and *WNT7B*) were associated with increased risk (HR range: 1.52–3.40, all p < 0.05, Fig. 3B). Three genes (*IGF1R, VRD, LEF1*) overlapped with DEG results comparing late versus no recurrence (Table S2). Cumulative incidence curves stratified by gene expression levels demonstrated patterns consistent with transition-specific hazard estimates. Although early recurrence was defined using a 5-year cutoff, most early recurrence events occurred within 4 years after surgery (Fig. 4).

### Transition-specific hazards: death outcomes

In the transition from no recurrence to death due to other causes, *IGF1* and *BORCS7* expression was associated with higher risk; a 1-SD increase corresponded to 2.24-fold (95%CI: 1.09–4.63) and 2.49-fold (95%CI: 1.05–5.89) higher risk, respectively (Table S3).

Conditional on patients who experienced recurrence, several genes were associated with BC-specific death. Among patients with early recurrence (12 of 14 recurred with metastatic disease), higher expression of *CD24*, *TMEM45B*, and *GRB7* was associated with increased risk of BC-specific death (HR range: 4.09–51.09, all p < 0.05), whereas *STAT1* and *CREBBP* expression was associated with lower risk (HR range: 3.9E-09–0.21, all p < 0.05, Table S4). No significant gene associations were observed for transitions from either early or late recurrence to other-cause death.

### Sensitivity analyses

Sensitivity analyses using alternative cutoffs for early versus late recurrence (3-year and 7-year) yielded results largely consistent with the primary 5-year definition. For the transition from no recurrence to early recurrence (Table S1), genes significant under the 5-year cutoff showed concordant effect sizes and directions under both alternative cutoffs, although the number of significant genes was reduced under the 3-year definition (*UBE2C, PTTG1, ORC6L, TOP2A, CDCA8, UBE2T*) and under the 7-year definition (*EZH2, CCNB1, MKI67, UBE2C, MELK, PTTG1, ORC6L, TOP2A, ANLN, CENPF, RRM2, CDCA8, PLCB1, CEP55, ITGB6, KIF2C, GPC4*, and *WNT11*). For the transition from no recurrence to late recurrence (Table S2), *PLCB1, IGF1R, WNT6*, and *WNT7B* remained significant across definitions, whereas *VRD* and *ITPR1* were directionally consistent under the 5-year model but not under the 7-year model. For the transition from early recurrence to BC-specific death, results were stable across all cutoffs (Table S4). Similar to the primary analysis, no significant associations were identified for recurrence-to-other-cause death transitions under alternative definitions (Table S5-S6).

### Pathway coherence through ORA and GSEA

**Figure 5:**
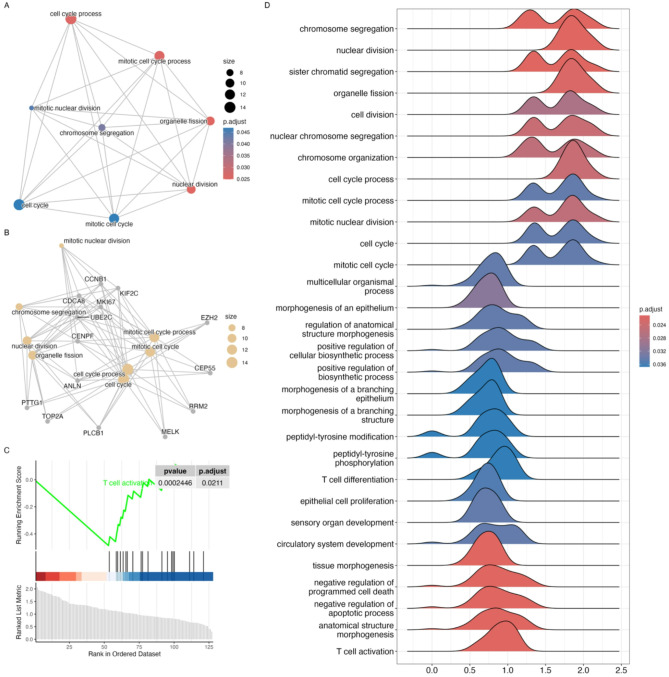
Pathway enrichment analyses of recurrence-associated genes. (A) Enrichment map of significantly overrepresented Gene Ontology (GO) Biological Process terms from over-representation analysis (ORA). Node size corresponds to the number of significant genes per category, node color reflects adjusted p-value, and edges represent semantic similarity among terms. (B) Gene-concept network linking enriched GO categories to significant genes identified in transition-specific Cox models. Orange nodes represent enriched GO terms, gray nodes represent genes, and edges indicate membership. (C) Gene set enrichment analysis (GSEA) enrichment curve for the pathway anatomical structure morphogenesis. The green line denotes the running enrichment score, vertical black bars indicate positions of pathway genes within the ranked list, and the lower panel shows the distribution of the ranked metric. (D) Ridgeplot summarizing significantly enriched GO terms from GSEA, with curves displaying enrichment score distributions colored by false discovery rate (FDR)-adjusted p-values. Abbreviations: p.adjusted, adjusted p-values

Genes significantly associated with the transition from no recurrence to early recurrence were not randomly distributed but clustered in cell cycle-related categories, including cell cycle process, mitotic cycle, nuclear division, and chromosome segregation (Fig. 5A). Gene-term networks highlighted *UBE2C*, *CCNB1*, *MKI67*, *CENPF*, *ANLN*, *KIF2C*, and *CDCA8* as central drivers mapping across multiple proliferative pathways (Fig. 5B).

Complementary GSEA using the full ranking of 128 genes demonstrated negative enrichment of pathways such as anatomical structure morphogenesis, with recurrence-protective genes concentrated at the protective end of the distribution (adjusted p = 0.001, Fig. 5C). Broader GSEA results identified enrichment of developmental and apoptotic processes, including cell development, programmed cell death, apoptosis, cell migration, and circulatory system development (Fig. 5D). No significant pathway enrichment was observed in other transition-specific models.

## Discussion

Our analyses consistently showed that early recurrence in ER + BC is associated with a concentrated set of proliferation- and mitosis-related genes. This pattern aligns closely with the biological foundations of several established commercial gene assays. The RS, for example, contains a dedicated proliferation module, including *MKI67, CCNB1, BIRC5*, and *MYBL2* (Fig. 3A), to which several of our strongest early-recurrence genes map directly (34, 35). The broader literature supports the view that proliferation-related genes capture important recurrence-associated biology. As reported by Buus et.al., signatures such as Risk of Recurrence (ROR) score, EndoPredict (EP), and BCI derive a greater proportion of their prognostic signal from proliferative biology (35). This provides biological context for our observed associations involving *UBE2C, EZH2, CCNB1*, and *MKI67* (Fig. 5A and 5B) as plausible prognostic genes linked to early recurrence risk (36). Notably, overlap with genes embedded in established assays should not be interpreted as evidence of clinical interchangeability or equivalence; rather, it highlights shared underlying biological programs that recur across independent platforms and signatures.

An important implication of our findings is the potential to inform development of focused temporally related multigene prognostic signatures. Existing signatures derive much of their prognostic value from proliferation-related modules, and recent efforts have explored compressed or reduced-gene versions of PAM50, Oncotype DX, and other assays. Our results highlight a reproducible set of proliferation and mitotic drivers—*UBE2C*, *EZH2*, *CCNB1*, *MKI67*, *CENPF*, *ANLN*, *KIF2C*, and *CDCA8*—that were consistently associated with early recurrence across multiple analytic frameworks. These high-information genes may represent candidates for constructing compact prognostic panels optimized for identifying patients at higher risk of early relapse. The substantial biological and genomic overlap with established assays supports future work incorporating cross-validation and external benchmarking to evaluate translational potential. Importantly, these associations were supported by consistent patterns observed across gene-level, transition-specific, sensitivity, and pathway-based analyses, suggesting that the identified genes reflect coherent biological programs rather than isolated statistical findings.

Our pathway analyses provide further biological context. Genes associated with the transition from no recurrence to early recurrence clustered in coherent pathways related to cell-cycle progression, mitotic spindle assembly, chromosome segregation, and nuclear division, with gene-term network analysis identifying *UBE2C, CCNB1, MKI67, CENPF, ANLN, KIF2C*, and *CDCA8* as central drivers mapping across multiple proliferative programs. Complementary GSEA showed negative enrichment of pathways such as anatomical structure morphogenesis, with recurrence-protective genes concentrated at the protective end of the ranked distribution (adjusted p = 0.001), while broader enrichment patterns highlighted coordinated involvement of developmental and apoptotic processes.

These pathway-level results do not establish mechanistic causality; instead, they indicate coordinated biological programs that are statistically associated with recurrence transitions. Together, these findings support a unified interpretation in which early recurrence is associated with heightened proliferative activity, a well-established feature of aggressive ER + BC (33, 37), accompanied by relative depletion of apoptotic signaling and differentiation programs that are more characteristic of less aggressive luminal phenotypes (38). In contrast, the absence of strong enrichment in other transition-specific models is consistent with evidence that late recurrence in ER + BC is less dependent on baseline proliferation and more strongly influenced by endocrine dependence, tumor and nodal burden, and microenvironmental cues (39). This contrast parallels the overlap between our early-recurrence genes and the proliferation modules embedded within assays such as Oncotype DX, PAM50, EndoPredict, and MammaPrint.

Our findings further converge with prior work showing that commercially used recurrence scores can be recapitulated using NanoString-based gene panels. Studies have demonstrated that Oncotype DX, EndoPredict, and Prosigna scores can be approximated from NanoString data and that their proliferation scores rely heavily on genes such as *ANLN, CDC20, CDCA1, CENPF, CEP55, KIF2C, KNTC2, MELK, MKI67, ORC6L, PTTG1, RRM2, TYMS, UBE2C*, and *UBE2T*—a list that substantially overlaps with the genes associated with early recurrence in our transition-specific models (36). These studies further showed that molecular-only, molecular-plus-clinical, and weighted approaches used by RS, EP, and ROR retain high concordance when computed from NanoString expression alone (36). Together, these observations reinforce the translational relevance of our results and indicate that the molecular patterns identified by our modeling framework recapitulate signals embedded within clinically validated assays.

Similarly, PAM50 incorporates several proliferation and mitotic regulators that appear among our significant genes, such as *ANLN, CCNB1, CDC20, CENPF, CEP55, EXO1, KIF2C, MELK, MMP11, ORC6, PTTG1*, and *RRM2* (40). Work on PAM50 compression further shows that accurate subtype classification can be achieved with reduced gene subsets that still include many of our top hits—*CCNB1, CENPF, CEP55, KIF2C, MELK, MKI67, PTTG1, RRM2, TYMS, UBE2C*, and *UBE2T*—demonstrating that these genes represent core, high-information genes across platforms (40). Additional studies show strong concordance between NanoString and RNA-seq quantification (41), increasing confidence in the robustness of our observations. The original PAM50 development work demonstrated substantial prognostic value beyond clinical features (9), and later long-term follow-up studies confirmed persistent prognostic utility over 15 years (42). Because our significant genes overlap with the PAM50 proliferation cluster (*CCNB1, CEP55, CENPF, ORC6L, KIF2C, PTTG1*, and *RRM2*), our results are consistent with, and extend, PAM50’s biological underpinnings into a recurrence-transition context (42).

Other commercial assays further reinforce the relevance of our findings. EndoPredict includes *UBE2C*, one of our strongest early-recurrence genes (Fig. 3A), as part of its 8-gene molecular score (43). The 70-gene MammaPrint assay is heavily enriched for cell-cycle and mitotic biological processes (44, 45), again mirroring the functional categories highlighted in our dataset. Our hit list also overlaps with Oncotype DX genes (*MKI67*, *CCNB1*, *BIRC5*, *MMP11*, *GRB7*) (34), additional PAM50 genes (19, 40), and the EndoPredict framework (43). Breast Cancer consists of two scores: 1) the ratio of two estrogen signaling genes [*HOXB13*/*L17BR* (H/I)] and 2) five genes that comprise the Molecular Grade Index (MGI)-*BUB1B*, *CENPA*, *NEK2*, *RACGAP1*, *RRM2*- representing the average score of a proliferation gene set. The H/I and MGI are able to predict long term benefits from ET (46). BCI is a clinically validated biomarker that predicts late recurrence in hormone positive BC patients. It has been used clinically to guide the administration of ET in patients who have remained recurrence-free for at least five years following surgery. It is utilized in both chemotherapy naive patients as well as those that have received either neoadjuvant or adjuvant chemotherapy. There is evidence however that it may be predictive of early recurrence in node negative patients (47), though this needs to be validated across different stages. This gene set has not yet been validated to discriminate early from late recurrence, though the genes included exhibit overlap with the pathways elucidated in our study. In our cohort, the high proportion of unavailable Oncotype scores precluded robust inferential analysis in this cohort. However, published real-world studies show that receipt of genomic testing is selective and is influenced by clinicopathologic factors, patient comorbidity and treatment preferences, clinician judgment, calendar era, and access-related factors. Accordingly, Oncotype results were not used as a formal covariate and should be interpreted as descriptive only in the present study. Collectively, these convergent lines of evidence suggest that the early-recurrence associations identified in our transition-specific analyses reflect well-established proliferative biology that has been repeatedly supported across platforms, assays, and clinical contexts.

These overlaps provide a framework to contextualize early versus late recurrence within ER + BC. ER + BC is characterized by a prolonged risk of relapse extending beyond 5 years, motivating our sensitivity analyses using 3- and 7-year thresholds (Table S1). These timepoints align with existing clinical practice and literature advocating for models that capture both early and late recurrence risk. However, in contrast to prominent work emphasizing the importance of long-term (>5-year) recurrence risk modeling (39), our dataset was more informative for short-term recurrence. Although recurrence prediction has been extensively studied, our transition-specific modeling adds temporal resolution by characterizing baseline molecular features associated with progression into early-recurrence states. In this cohort, baseline proliferative programs were more strongly associated with transitions to early recurrence than with late recurrence, highlighting temporal heterogeneity in recurrence-related molecular patterns. This nuance supports the interpretation that early relapse in ER + BC is dominated by highly proliferative tumors and may reflect biological processes distinct from those governing later relapse.

Several strengths of this study merit emphasis. First, the use of a semi-Markov multi-state modeling framework aligns naturally with the clinical trajectory of ER + BC and enables explicit modeling of transitions from no recurrence to early and late recurrence, as well as post-recurrence outcomes. This structure allowed us to extract time-dependent molecular associations from a modest-sized, well-characterized institutional cohort and to distinguish baseline features associated with progression into early-recurrence states without imposing a single proportional hazards assumption over the entire follow-up. The convergence of transition-specific inference with coherent pathway enrichment provides internal biological support for our findings, particularly for proliferative and mitotic programs, despite the limited sample size. Our results further demonstrate that biologically meaningful patterns can be recovered from focused, high-quality institutional datasets when paired with modeling frameworks that respect disease dynamics.

At the same time, several important limitations should be acknowledged. The cohort size of 79 patients limits statistical power, particularly for later transitions with sparse events, and constrains the generalizability of our effect estimates, as the sample may not fully represent the broader community distribution. In addition, only single baseline tumor samples obtained at surgery were available for each patient. While baseline expression may reflect initial risk propensity, this design does not capture temporal changes in gene expression at the time of recurrence. Our analyses detail associations and cannot establish etiologic causality.

While we observed that certain genes were statistically significant for early but not late recurrence, we did not formally test heterogeneity of effects across recurrence timing; thus, such patterns should be interpreted cautiously. The absence of matched commercial assay scores in our cohort precludes direct benchmarking against established signatures. Although the cohort included both luminal A and luminal B tumors, the predominance of proliferation-associated signals and the substantial representation of luminal B cases suggest that the observed associations may be most applicable to biologically aggressive ER+ subgroups. Finally, we were unable to account for post-surgical treatment duration, adherence, or therapy switching, and thus cannot disentangle intrinsic tumor biology from treatment-modified recurrence risk. External validation in larger, independent cohorts will be essential to confirm the robustness and reproducibility of these findings.

## Conclusions

In summary, by embedding gene expression profiling within a semi-Markov multi-state framework, we demonstrate that early recurrence in ER + BC is consistently associated with a compact set of proliferation- and mitosis-related genes that overlap extensively with modules embedded in clinically validated prognostic assays. Our results suggest that transition-specific modeling can disentangle temporal patterns of molecular association and provide complementary insight into recurrence timing beyond conventional survival analyses. While further validation is required, these findings offer a foundation for refining recurrence-timing biomarkers and motivate future studies integrating dynamic modeling with larger, externally validated genomic cohorts.

## Figures and Tables

**Table 1:** Demographic and clinical characteristics of the ER+ breast cancer cohort by recurrence type

Characteristics	Total (N = 79)	Breast cancer recurrence type			P-value^a^
		No (n = 56)	Early (n = 14)	Late (n = 9)	
**Race and ethnicity**					
White, non-Hispanic	42 (53.16%)	27 (48.21%)	10 (71.43%)	5 (55.56%)	0.48
Asian, non-Hispanic	17 (21.52%)	12 (21.43%)	2 (14.29%)	3 (33.33%)	
Hispanic	17 (21.52%)	15 (26.79%)	1 (7.14%)	1 (11.11%)	
Other or unknown	3 (3.8%)	2 (3.57%)	1 (7.14%)	0 (0%)	
**Age at diagnosis**					
≤50	26 (32.91%)	18 (32.14%)	6 (42.86%)	2 (22.22%)	0.73
51–65	32 (40.51%)	24 (42.86%)	5 (35.71%)	3 (33.33%)	
66+	21 (26.58%)	14 (25%)	3 (21.43%)	4 (44.44%)	
**Body mass index at diagnosis**					
<24	21 (26.58%)	13 (23.21%)	4 (28.57%)	4 (44.44%)	0.59
24–30	35 (44.3%)	27 (48.21%)	6 (42.86%)	2 (22.22%)	
31+	23 (29.11%)	16 (28.57%)	4 (28.57%)	3 (33.33%)	
**Diabetes at diagnosis**					
No	70 (88.61%)	49 (87.5%)	13 (92.86%)	8 (88.89%)	0.99
Yes	9 (11.39%)	7 (12.5%)	1 (7.14%)	1 (11.11%)	
**Tumor size**					
≤3	62 (78.48%)	44 (78.57%)	11 (78.57%)	7 (77.78%)	0.99
3.1+	17 (21.52%)	12 (21.43%)	3 (21.43%)	2 (22.22%)	
**Cance stage**					
I/II	58 (73.42%)	42 (75%)	11 (78.57%)	5 (55.56%)	0.40
III	21 (26.58%)	14 (25%)	3 (21.43%)	4 (44.44%)	
**Survival status**					
Breast Cancer	6 (7.59%)	0 (0%)	6 (42.86%)	0 (0%)	<0.001
Others	8 (10.13%)	4 (7.14%)	2 (14.29%)	2 (22.22%)	0.33
**Oncotype score**					
Low (0–17)	21 (26.58%)	18 (32.14%)	1 (7.14%)	2 (22.22%)	0.04
Intermediate (18–30)	16 (20.25%)	11 (19.64%)	4 (28.57%)	1 (11.11%)	
High (≥31)	5 (6.33%)	2 (3.57%)	3 (21.43%)	0 (0%)	
Score unavailable	37 (46.84%)	25 (44.64%)	6 (42.86%)	6 (66.67%)

a Fisher’s exact test was used to assess group differences in categorical variables, and Gray’s test was applied to compare survival status across recurrence groups.

## Data Availability

The data and code analyzed in this study are not publicly available due to patient privacy but deidentified data are available from the corresponding author on reasonable request.
